# The relationship between behavioral activation and burnout in a community setting: the mediating role of acceptance-based action, automatic negative thought, and self-efficacy

**DOI:** 10.3389/fpsyg.2025.1585047

**Published:** 2025-08-01

**Authors:** Hyewon Yeo, Jini Tae, Yoonhyoung Lee, Youngeun Kim, Wonhye Lee

**Affiliations:** ^1^Department of Psychiatry, Samsung Medical Center, Seoul, Republic of Korea; ^2^School of Humanities and Social Sciences, Gwangju Institute of Science and Technology, Gwangju, Republic of Korea; ^3^Department of Psychology, Yeungnam University, Daegu, Republic of Korea; ^4^Korea Brain Research Institute, Daegu, Republic of Korea

**Keywords:** burnout, behavioral activation, acceptance-based action, automatic negative thought, self-efficacy, structural equation model (SEM)

## Abstract

**Introduction:**

Burnout, a work-related syndrome, considerably overlaps with depression. Despite its negative impact on modern society, the underlying mechanisms and effective interventions remain unclear. This study explores the effects of behavioral activation (BA) on burnout and investigates potential mediators within this relationship.

**Method:**

Participants consisted of 471 Korean adults aged 20 to 69, recruited from the community through a research panel. They completed a self-administered online survey via personal computers or mobile devices. The survey included self-report measures such as the Behavioral Activation for Depression Scale-Short Form (BADS-SF), the Acceptance and Action Questionnaire-II (AAQ-II), the Self-Efficacy (SE).

**Results:**

Path analysis results indicated that higher behavioral activation levels were associated with increased acceptance-based action and self-efficacy, as well as reduced automatic negative thoughts. Additionally, greater acceptance-based action and self-efficacy were linked to lower burnout levels, whereas more frequent automatic negative thoughts were associated with higher burnout levels. Furthermore, structural equation modeling revealed that the relationship between behavioral activation and psychological burnout was fully mediated by these three variables.

**Discussion:**

This study demonstrated a significant relationship between behavioral activation and burnout symptoms, with acceptance-based action, automatic negative thoughts, and self-efficacy acting as mediators. These findings offer valuable insights for developing effective treatment protocols for burnout within the BA framework.

## Introduction

Burnout syndrome encompasses emotional exhaustion, cynicism, and professional inefficacy, all of which significantly affect job satisfaction and overall wellbeing (Maslach et al., 2001). The recent framework of burnout clarified energetic, motivational, emotional, and cognitive impairments, emphasizing cognitive and emotional self-regulation difficulties that were previously subsumed under the concept of exhaustion (Schaufeli et al., 2020). Occupational stressors, including work overload, emotional labor, lack of autonomy, role conflict, insufficient social support, and long working hours, trigger burnout (Edú-Valsania et al., 2022). Burnout is associated with a range of negative consequences, including depression, insomnia, cognitive impairments, and an increased risk of suicide (Daloee et al., 2020; Gavelin et al., 2022; Kuriyama, 2023; Menon et al., 2020; Nguyen et al., 2025). Burnout can lead to avoidance behaviors such as increased absenteeism and a higher intention to leave the job (Lee et al., 2023).

Burnout demonstrates considerable overlap with depression (Tavella et al., 2023), particularly in symptoms such as exhaustion and unhedonia. Many empirical studies have reported a positive correlation between depression and burnout (Ahola et al., 2014; Bianchi et al., 2021), further supported by evidence of DNA methylation observed in stress-related disorders such as depression (Bakusic et al., 2021). Burnout has also been conceptualized as a form of occupational depression, characterized by depressive symptoms, specifically resulting from work-related stress (Bianchi and Sowden, 2022).

Discrepancies between work demands and individual resources can exacerbate burnout, highlighting the importance of coping capability in managing work stress (Alarcon, 2011). While some individuals develop burnout under stressful work conditions, others remain resilient. Specifically, cognitive style, behavioral pattern, and self-efficacy are associated with individual coping capabilities in response to work stress.

**Cognitive style**. Burned-out individuals often exhibit negative cognitive biases, such as dysfunctional attitudes, rumination, and pessimistic attribution, similar to those observed in depression (Bianchi and Schonfeld, 2016). Negative memory biases, characterized by an increased recall of negative words and a decreased recall of positive words, were associated with burnout (Bianchi et al., 2020). Moreover, a greater tendency to endorse negative information over positive information when interpreting emotional stimuli has been observed in individuals experiencing burnout (Bianchi and da Silva Nogueira, 2019; Bianchi et al., 2018). Negative cognitive styles, such as negative automatic thoughts and rumination, contribute to burnout (Shigematsu et al., 2024).

**Behavioral pattern**. The capacity to accept distressing thoughts and emotions while committing to value-driven action enables one to face challenging situations rather than avoiding stressors (Hayes, 2019). Committing to valued actions is associated with job-crafting behavior and recovery processes that help build the necessary psychological and emotional resources to cope with ongoing job demands (Bakker and De Vries, 2021). On the other hand, avoidance behaviors, which provide temporary relief from discomfort but ultimately reinforce stress and emotional exhaustion, thereby perpetuating burnout (Noone and Hastings, 2011).

**Self-efficacy**. A large number of studies across various job populations and countries have consistently demonstrated a negative relationship between self-efficacy and burnout (Kim and Burić, 2020; Liu and Aungsuroch, 2019; Shoji et al., 2016). Social self-efficacy not only helps prevent burnout and emotional impairments but also reduces interpersonal strain at work (Brunsting et al., 2024).

Evidence-based interventions such as mindfulness-based Cognitive Behavioral Therapy (Paudel et al., 2025) and Acceptance and Commitment Training (Szarko et al., 2022; Towey-Swift et al., 2023) have been shown to significantly reduce burnout symptoms. However, their effectiveness has produced inconsistent results depending on study design, intervention type, and participant characteristics (Madigan et al., 2024). In addition, a clear distinction between interventions aimed at managing relatively mild work-related stress and those targeting clinical burnout has yet to be consistently established (Ahola et al., 2017).

Given the conceptual overlap between depression and burnout, we propose that the mechanisms of change in behavioral activation (BA) may offer valuable insights for burnout interventions. Although empirical evidence for the use of BA in burnout is still limited, its core principles—such as reducing avoidance and increasing engagement in meaningful activities—are closely aligned with strategies shown to alleviate depressive symptoms (Nagy et al., 2020). These mechanisms may also be relevant in occupational contexts, as proactive behaviors aimed at maintaining or enhancing job control have been associated with lower levels of burnout (Hendrikx et al., 2024). Honarvar et al. (2023) reported a decrease in burnout subcategories—emotional exhaustion, depersonalization, and reduced personal accomplishment—following a BA intervention. Establishing the relevance of BA mechanisms could advance theoretical understanding and support the development of more effective interventions for burnout.

This study examines the effect of behavioral activation on burnout. Specifically, we investigate the mediating roles of cognitive style (automatic negative thought), behavioral patterns (acceptance-based action), and self-efficacy. Based on this framework, we propose the following hypotheses:

- H_1_: Behavioral activation is negatively associated with burnout.
- H_2_: Automatic negative thought mediates the relationship between behavioral activation and burnout.
- H_3_: Acceptance-based action mediates the relationship between behavioral activation and burnout.
- H_4_: Self-efficacy mediates the relationship between behavioral activation and burnout.

## Materials and methods

### Participants and procedures

Participants were recruited through Southernpost, a professional research panel company in South Korea (https://southernpost.co.kr/). All participants were native Korean speakers and registered members of the company's research panel. The company randomly selected individuals aged 20 to 69 from their panel and sent them an invitation explaining the purpose of the study and requesting their participation. Those who expressed interest received a link to the survey, which they completed via either web or mobile platforms.

The study was approved by the Institutional Review Board at Yeungnam University. Informed consent was obtained from all participants prior to the start of the survey. Participants first completed a brief demographic questionnaire, followed by a set of self-report measures presented in random order: the Behavioral Activation for Depression Scale–Short Form (BADS-SF), the Acceptance and Action Questionnaire-II (AAQ-II), the Self-Efficacy (SE) Scale, the Automatic Thoughts Questionnaire–Negative (ATQ-N), and the Korean version of the Burnout Assessment Tool (K-BAT). No additional instructions were provided beyond those included in each measure.

A total of 500 native Korean adults between the ages of 20 and 69 completed the survey. After excluding 29 responses due to low-quality or insincere answers, the final sample consisted of 471 participants.

### Measures

#### Behavioral Activation for Depression Scale-Short Form

The Korean version of BADS-SF consisted of 9 items, grouped into two subscales: activation (6 items) and Avoidance (3 items) (Kim et al., 2024). Participants were instructed to carefully read each statement and rate how accurately it described their experiences over the past week, including the day of assessment. Responses were recorded on a seven-point scale ranging from 0 (not at all) to 6 (completely). For scoring, items 1, 6, 7, and 8 were reverse-coded, after which all item scores were summed to calculate the total score. Higher total scores indicated greater behavioral activation. Internal consistency, as measured by Cronbach's α, was 0.78 in the current sample.

#### Acceptance and Action Questionnaire-II

Heo et al. (2009) translated the original Acceptance and Action Questionnaire-II (AAQ-II; Bond et al., 2011) into Korean. The AAQ-II consists of 10 items, with participants rating each item on a 7-point Likert scale ranging from 1 (never true) to 7 (always true). Higher total scores indicate a higher level of acceptance-based action. Bond et al. (2011) reported an internal consistency (Cronbach's α) of.84 for the original scale. Heo et al. (2009) validated the Korean version and reported a Cronbach's α of 0.85. Consistent with Heo et al. (2009), the internal consistency in the current study was also 0.84.

#### Self-Efficacy Scale

Hong (1995) translated the original Self-Efficacy (SE) Scale (Sherer et al., 1982) into Korean, and we utilized this Korean version in our study. The SE Scale consisted of 23 items divided into two subscales: General Self-Efficacy (17 items) and Social Self-Efficacy (6 items). Participants rated each item on a 5-point Likert scale ranging from 1 (Strongly Disagree) to 5 (Strongly Agree). To calculate the total score, all item scores were summed, with higher total scores indicating greater self-efficacy. A reliability estimate of 0.90 (Cronbach's α) was obtained for the scale.

#### Automatic Thought Questionnaire-N

The ATQ-N is a self-report questionnaire designed to measure how frequently an individual experiences negative thoughts in daily life. It was originally developed by Hollon and Kendall (1980) and later translated into Korean by Kwon et al. (1994). This study utilized the Korean version of the ATQ-N, which consists of 30 items. Participants rated each item on a 5-point Likert scale, ranging from 1 (Not at all) to 5 (Always). The total score was calculated by summing all item scores, with higher scores indicating a greater frequency of negative thoughts. The original authors, Hollon and Kendall (1980), reported an internal consistency (Cronbach's α) of 0.96. Similarly, Yang et al. (2005), who applied the scale to a sample of adolescents, also found an internal consistency of 0.96. In the present study, which involved Korean adults aged 20 to 69, the internal consistency was 0.98.

#### Korean version of Burnout Assessment

K-BAT (Cho, 2020) is designed to measure the burnout level of the participants based on their self-report. The total number of items were 22 and items were grouped into 4 subscales, exhaustion (8 items), mental distance (4 items), impaired cognitive control (5 items) and impaired emotional control (5 items). Since the current study mainly focused on cognitive and emotional control impairment, only 10 items were utilized in this study. Participants were asked to check how often they experienced certain events using the 5-Likert scale ranging from 1 (Not at all) to 5 (Always). This measure yielded a Cronbach's alpha of 0.91 in the current study.

### Analysis

All psychological scales used different Likert-type response formats. BADS-SF and AAQ-II employed a 7-point Likert scale, while the remaining questionnaires used a 5-point scale. For each measure, total scores were calculated by summing item responses, and these totals were used independently in the correlation and structural equation modeling (SEM) analyses. Because both Pearson's correlation and SEM estimate standardized relationships (e.g., standardized coefficients and covariances), differences in response formats do not affect the estimation or interpretation of the results. These analyses are based on the variances and covariances among variables, rather than the raw scale metrics.

Data analysis was conducted using IBM SPSS (v.21.0). First, Pearson's correlation coefficients were computed to examine the relationships among the BADS-SF, AAQ-II, SE Scale, ATQ-N, and K-BAT. Subsequently, SEM was performed using IBM AMOS (v.21.0) to investigate whether behavioral activation influenced burnout indirectly through acceptance-based action, self-efficacy, and automatic negative thoughts.

## Results

### Descriptive statistics

The descriptive statistics of the sample are presented in Table 1. The proportion of male and female participants was equal. Most participants had attended university (69.85%), in addition 12.95% had completed postgraduate education. The mean scores (and standard deviations) for the scales were as follows: BADS-SF, 30.88 (SD = 7.21); AAQ-II, 44.62 (SD = 8.71); SE, 76.11 (SD = 11.73); ATQ-N, 59.33 (SD = 25.07); and BAT (cognitive and emotional control subscales only), 22.79 (SD = 7.46).

**Table 1:** Demographic characteristics of the sample.

**Variable**	***N* (%)**
**Sex**
Male	235 (50.0)
Female	236 (50.0)
**Age (yr)**
M	44.76
SD	13.42
**Education**
Middle school graduate	1 (0.21)
High school graduate	80 (16.99)
University	329 (69.85)
Postgraduate	61 (12.95)

### Correlations

The correlation results are presented in Table 2. All correlation coefficients between the variables were statistically significant. The BADS-SF showed significant positive correlations with AAQ-II (*r* = 0.58, *p* < 0.001) and SE, while demonstrating negative correlations with ATQ-N (*r* = −0.556, *p* < 0.001) and K-BAT (*r* = −0.574, *p* < 0.001). AAQ-II was positively correlated with SE (*r* = 0.575, *p* < 0.001) and negatively correlated with both ATQ-N (*r* = −0.64, *p* < 0.001) and K-BAT (*r* = −0.75, *p* < 0.001). Similarly, SE exhibited significant negative correlations with ATQ-N (*r* = −0.591, *p* < 0.001) and K-BAT (*r* = −0.589, *p* < 0.001). Finally, ATQ-N was positively correlated with K-BAT (*r* = 0.696, *p* < 0.001).

**Table 2:** Descriptive statistics (mean, standard deviation, skewness and kurtosis) and correlation between variables.

**Variable**	**1**	**2**	**3**	**4**	**5**
1. BADS-SF					
2. AAQ-II	0.58^***^				
3. SE	0.673^***^	0.575^***^			
4. ATQ-N	−0.556^***^	−0.64^***^	−0.591^***^		
5. K-BAT	−0.576^***^	−0.75^***^	−0.589^***^	0.696^***^	
M (SD)	30.88 (7.21)	44.62 (8.71)	76.11 (11.73)	59.33 (25.07)	22.79 (7.46)
Skewness	−0.062	−0.304	0.169	1.141	0.556
Kurtosis	0.459	0.215	−0.13	0.653	0.176

BADS-SF, Behavioral activation for depression scale-short form; AAQ-II, Acceptance and Action Questionnaire II; SE, Self-Efficacy Scale; ATQ-N, Automatic Thought Questionnaire-N; K-BAT, Korean version of Burnout Assessment.

^***^p < 0.001.

### Structural equation model

**Figure 1:**
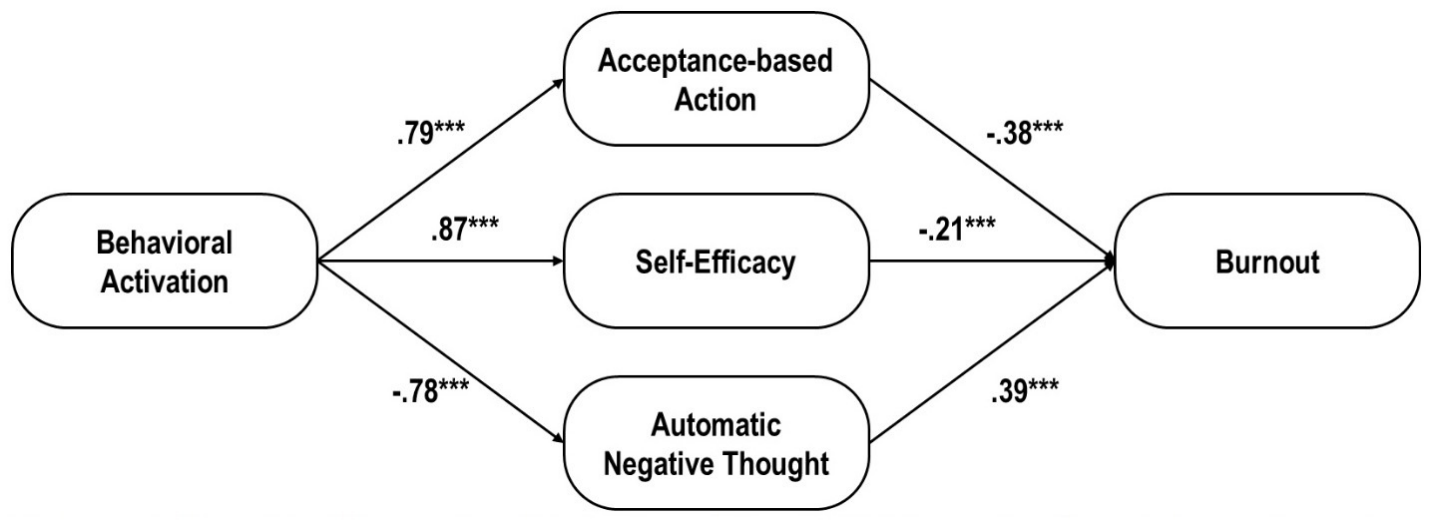
Structural Equation Modeling of themediating role of acceptance-based action, self-efficacy, and automatic negative thought between behavioral activation and burnout. ^***^p < 0.001.

We analyzed the structural model to examine whether acceptance-based action, self-efficacy, and automatic negative thought mediate the effect of behavioral activation on psychological burnout. As hypothesized, the structural model demonstrated a good fit to the data, with fit indices as follows: NFI = 0.951, CFI = 0.959, IFI = 0.960, TLI = 0.939, RMSEA = 0.098, and RMR = 0.04. Figure 1 illustrates the proposed model depicting the relationship between behavioral activation levels and burnout levels.

The path analysis results, presented in Table 3, reveal that the standardized path coefficients of behavioral activation were significant for acceptance-based action (β = 0.799, *p* < 0.001), self-efficacy (β = 0.872, *p* < 0.001), and automatic negative thought (β = −0.789, *p* < 0.001). These results indicate that higher behavioral activation levels were associated with increased acceptance-based action and self-efficacy, as well as reduced automatic negative thought. Furthermore, the standardized path coefficients of acceptance-based action (β = −0.381, *p* < 0.001), self-efficacy (β = −0.211, *p* < 0.001), and automatic negative thought (β = 0.395, *p* < 0.001) were significant in predicting burnout levels. Specifically, increased acceptance-based action and self-efficacy were associated with lower burnout levels, whereas more frequent automatic negative thoughts were linked to higher burnout levels.

**Table 3:** Path analysis results for the proposed model.

**Path**	**Estimate**		**S.E**	**Critical ratio (CR)**
	**Unstandardized estimate (B)**	**Standardized estimate** ***(**β**)***		
Behavioral activation → Acceptance-based Action	2.209	0.799	0.175	12.092^***^
Behavioral activation → Self-efficacy	0.613	0.872	0.065	9.369^***^
Behavioral activation → Automatic negative thought	−0.992	−0.789	0.082	−12.092^***^
Acceptance-based action → Burnout	−0.15	−0.381	0.019	−7.828^***^
Self-efficacy → Burnout	−0.332	−0.211	0.099	−3.362^***^
Automatic negative thought → Burnout	0.346	0.395	0.045	7.63^***^

^***^p < 0.001.

To examine the indirect effects of acceptance-based action, self-efficacy, and automatic negative thought pathways on the relationship between behavioral activation and psychological burnout, phantom variables were utilized. The analysis revealed that the indirect effects of the acceptance-based action pathway (*B* = −0.331, *p* < 0.01), self-efficacy pathway (*B* = −0.203, *p* < 0.01), and automatic negative thought pathway (*B* = −0.343, *p* < 0.01) on the relationship between behavioral activation and psychological burnout were statistically significant (Table 4).

**Table 4:** Mediation effect on final mode.

**Path**	**Unstandardized estimate (B)**	**S.E**	**Bootstrapping bias-corrected percentile method**	
			**Lower**	**Upper**
Behavioral activation → Acceptance-based action → Burnout	−0.331	0.066	−0.475	−0.22
Behavioral activation → Self-efficacy → Burnout	−0.203	0.087	−0.422	−0.07
Behavioral activation → Automatic negative thoughts → Burnout	−0.343	0.068	−0.499	−0.231

## Discussion

Our structural equation model confirmed all proposed hypotheses, suggesting that behavioral activation may be an effective mechanism for alleviating burnout. As hypothesized, the mediating roles of automatic negative thought, acceptance-based action, and self-efficacy were supported. Consistent with previous research, the positive roles of acceptance-based action and self-efficacy, along with the negative mediating role of automatic negative thoughts, highlight both risk and protective factors relevant to burnout intervention. Taken together, these results offer empirical support for the hypothesized model and highlight the multi-faceted pathways through which behavioral activation can influence burnout.

Individuals experiencing burnout often show a reduction in overall activity levels (Naczenski et al., 2017; Taylor et al., 2022), which reduces opportunities for positive reinforcement from work-related accomplishments. Behavioral activation can be a source of not only external rewards (e.g., work achievements) but also intrinsic rewards (e.g., feelings of autonomy and competence, or self-efficacy). Self-efficacy, in particular, is known to activate a neutral reward system and make activities intrinsically rewarding (Blain and Sharot, 2021). As behavioral activation increases goal-directed behaviors, it enhances individuals' self-efficacy. Then, they may become more sensitive to intrinsic rewards, amplifying the positive effects of behavioral activation and eventually mitigating burnout. Self-efficacy not only serves as a protective factor against burnout but also acts as an intrinsic reward itself, further reinforcing behavioral activation.

Acceptance-based action refers to psychological flexibility in pursuing one's values, even in the presence of challenging situations. Regardless of the contingencies of reinforcement or punishment, individuals with higher acceptance-based action can maintain a goal-directed behavior and remain committed to their values. Additionally, when confronted with internal experiences such as anxiety and self-doubt, acceptance-based action prevents avoidance and maintains meaningful behaviors aligned with their core values (Bond et al., 2013). By promoting goal-directed behavior and reducing avoidance tendencies, acceptance-based action may ultimately contribute to reducing burnout. While a recent review of the treatment process of ACT reported inconsistent associations between value and burnout (Towey-Swift et al., 2023), our findings underscore the potential role of acceptance-based action in sustaining the effects of behavioral activation.

Negative cognitive patterns, such as dysfunctional attitudes, rumination, pessimistic attributional style, and perfectionism, have been strongly associated with burnout (Bianchi and Schonfeld, 2016; Philp et al., 2012). Among these, perfectionism in burnout is particularly associated with procrastination (Khadija and Azim, 2023), a form of avoidance behavior that reduces exposure to workplace stressors. Avoidance behavior reinforces self-critical rumination and impairs an individual's ability to tolerate failure (van der Kaap-Deeder et al., 2016). Through behavioral activation, individuals increase their exposure to positive reinforcement, experience small successes that challenge negative expectations, and weaken dysfunctional beliefs, reducing workplace burnout.

Several limitations should be considered. First, the study relies on self-report measures, which may introduce bias. Future research should incorporate observational, biological, or experimental methods to enhance validity. Second, although our sample included a wide age range (20–69 years) from community settings, all participants were recruited through a recruiting company, with only gender and age information available. While this broad age range enhances generalizability, the exclusive use of web or mobile-based surveys suggests that the sample may be biased toward individuals with higher digital literacy and cultural capital. Consequently, it remains unclear whether the observed relationships extend to populations with lower educational attainment or limited access to digital resources. Future studies should include more diverse samples that account for variations in educational background and technological accessibility. Third, the absence of a control group makes it difficult to determine whether the observed effects result from behavioral activation itself or other confounding factors, such as spontaneous recovery or external influences. Fourth, the cross-sectional design of this study prevents causal inferences. Longitudinal research is needed to clarify the temporal dynamics between behavioral activation and burnout.

This is the first study to suggest behavioral activation as a potential mechanism for reducing burnout and to examine the mediating roles of acceptance-based action, automatic negative thought, and self-efficacy. In addition to the primary focus on increasing activity, our findings highlight the importance of cognitive restructuring, psychological flexibility, and self-efficacy in burnout-specific interventions. These results support the development of a standardized BA protocol incorporating problem-solving skills, mindfulness training, and cognitive restructuring techniques to enhance recovery from burnout. Importantly, the mechanisms identified in this study—particularly acceptance-based action and self-efficacy—are conceptually aligned with key constructs in major occupational stress models. The Job Demands–Resources (JD-R) theory, for example, explains burnout as the result of an imbalance between job demands and resources, where personal resources such as self-efficacy and psychological flexibility act as critical buffers (Bakker and De Vries, 2021). Similarly, the Conservation of Resources (COR) theory emphasizes the importance of preserving and replenishing psychological resources in preventing stress-related outcomes (Hobfoll et al., 2015). From this perspective, behavioral activation can be viewed as a mechanism for mobilizing and restoring such resources—through increased goal-directed behavior, intrinsic reward, and values-driven engagement. By situating behavioral activation within these well-established frameworks, future research can contribute to a more integrated theoretical understanding of how individual-level interventions mitigate the effects of systemic work stressors. Comparative trials would clarify the specificity of BA mechanisms and enhance the practical relevance of burnout interventions across occupational contexts.

Future research should investigate these mechanisms using longitudinal or experimental designs to more firmly establish causality. It is also important to assess the effectiveness of burnout-specific BA protocols across occupational groups and cultural contexts. A next step will be to develop and clinically validate standardized BA protocols to determine their efficacy and real-world applicability. Moreover, individual characteristics—such as proactive personality traits, motivational orientation, or emotion regulation capacity—should be considered as potential moderators of intervention success.

## Data Availability

The original contributions presented in the study are included in the article/Supplementary material, further inquiries can be directed to the corresponding author.
