# Supplementary material for: The relationship between behavioral activation and burnout in a community setting: the mediating role of acceptance-based action, automatic negative thought, and self-efficacy
Source: Front Psychol. 2025 Aug 1;16:1585047. doi: 10.3389/fpsyg.2025.1585047 (PMC12354651; doi:10.3389/fpsyg.2025.1585047)
Supplement: Supplementary file 1 [file Table_1.DOCX]

**Supplementary Materials**

**Supplementary Table 1.**

Behavioral activation for depression scale-short form (BADS-SF)^1^

| 1 | 꼭 해야 하는데 하지 않은 일들이 있다. |
| --- | --- |
| 2 | 내가 한 일의 종류와 양에 만족한다. |
| 3 | 나는 광범위하고 다양한 활동들에 참여했다. |
| 4 | 나는 어떤 활동 혹은 상황에 참여할지에 대해 좋은 선택을 했다. |
| 5 | 나는 활동적인 사람이고 내가 정한 목표를 완수했다. |
| 6 | 내가 한 일의 대부분은 불쾌한 상황을 피하거나 벗어나기 위함이었다. |
| 7 | 나의 걱정거리에 대해 곱씹느라 많은 시간을 소모했다. |
| 8 | 나는 불쾌한 감정으로부터 주의를 분산시키기 위한 활동에만 참여했다. |
| 9 | 나는 즐거운 일을 했다. |

^1^ Kim, Y. E., Tae, J.E. Lee, Y.H., Park, K.H., Lee, W.H. (2024). A Reliability and Validity of the Korean Version of Behavioral Activation for Depression Scale-Short Form: A Community Sample of Adults. The Korean Society of Stress Medicine, 32(4), 214-220

Acceptance and Action Questionnaire-Ⅱ (AAQ-Ⅱ)^2^

| 1 | 뭔가 부정적인 것을 기억하게 되어도 괜찮다. |
| --- | --- |
| 2 | 나의 고통스러운 과거 경험과 기억이 내가 추구하는 삶을 살기 어렵게 한다. |
| 3 | 내 감정에 대해 겁이 난다. |
| 4 | 스스로의 걱정과 감정을 통제하지 못할 까봐 겁이 난다. |
| 5 | 나의 고통스러운 경험이 충만한 삶을 살기 어렵게 한다. |
| 6 | 나의 삶에 대해 통제력을 잘 발휘하고 있다. |
| 7 | 내 삶에서 감정이 문제를 야기한다. |
| 8 | 대부분의 사람들은 나에 비해 자신의 삶을 잘 영위해가고 있는 것 같다. |
| 9 | 염려가 나의 성공을 가로막는다. |
| 10 | 나의 생각과 감정은 내가 어떤 삶을 살아가고 싶은지에 별 영향을 주지 않는다. |

^2^ Heo, J., Choi, M., & Jin, H. (2009). Study on the reliability and va lidity of a Korean translated Acceptance and Action Questionnaire-II. Korean Journal of Counseling and Psychotherapy, 21, 861-878.

Self-Efficacy (SE) Scale^3^

| 1 | 나는 어떤 일이든 계획대로 수행할 수 있다. |
| --- | --- |
| 2 | 나는 일을 해야 할 때 바로 일을 시작하지 못하는 문제점이 있다.* |
| 3 | 어떤 일을 처음에 잘못 했더라도 될 때까지 해 본다. |
| 4 | 나는 중요한 목표를 설정하면 성취할 수 있다. |
| 5 | 나는 어떤 일을 끝마치기도 전에 포기한다.* |
| 6 | 나는 어려운 일에 부딪히는 것을 피한다.* |
| 7 | 나는 어떤 일이 너무 복잡해 보이면 해 볼 시도조차 안 한다.* |
| 8 | 별로 유쾌하지 않은 어떤 일을 할 때 나는 그것을 끝마칠 때까지 반드시 한다. |
| 9 | 나는 뭔가 할 일이 있을 때 바로 그 일을 시작한다. |
| 10 | 새로운 어떤 일을 배우려고 시도할 때 처음에 성공할 것 같지 않으면 바로 포기한다.* |
| 11 | 예기치 못한 문제가 일어나면 나는 잘 대처할 수 없다.* |
| 12 | 나는 새로운 일이 너무 어려우면 배우려고 하지 않는다.* |
| 13 | 실패는 나로 하여금 더 열심히 노력하도록 만들 뿐이다. |
| 14 | 나는 어떤 일을 할 수 있는지 능력에 불안함을 느낄 때가 있다.* |
| 15 | 나는 자신감이 있다. |
| 16 | 나는 쉽게 포기한다.* |
| 17 | 나는 인생에 부딪히는 거의 모든 문제들을 다룰 능력이 없는 것 같다.* |
| 18 | 새 친구를 사귀는 일은 내게 너무 어려운 일이다.* |
| 19 | 나는 어떤 사람이 보고 싶으면 그 사람이 와주기를 기다리는 대신에 내가 먼저 간다. |
| 20 | 내가 관심이 가는 어떤 사람이 사귀기 어려운 사람이라도 나는 사귀는 것을 금방 포기한다.* |
| 21 | 첫눈에 호감이 가지 않는 사람이라고 해도 나는 그 사람과 사귀는 것을 쉽게 그만두지 않는다. |
| 22 | 나는 사교적 모임에서 내 자신을 어찌해야 하는지 잘 모르겠다.* |
| 23 | 지금의 내 친구들은 사교성 덕분에 사귀었다. |

^3^ Hong. (1995). Relationship of perfectionism, self-efficacy, and depression. Master degree, Ewha Womans Univerisy,

Automatic Thought Questionnaire-N (ATQ-N)^4^

| 1 | 세상이 나를 힘들게 하는 것처럼 느껴진다. |
| --- | --- |
| 2 | 나는 쓸모없는 사람이다. |
| 3 | 도대체 왜 나는 성공할 수 없는 것일까? |
| 4 | 아무도 나를 이해해주지 않는다. |
| 5 | 나는 종종 사람들을 실망시켜 왔다. |
| 6 | 나는 내가 하는 일을 계속할 수 없을 것 같다. |
| 7 | 나는 더 나은 사람이 되고 싶다. |
| 8 | 나는 너무 나약하다. |
| 9 | 내 인생은 내가 원하는 대로 흘러가고 있지 않다. |
| 10 | 나는 내 자신에 대해 매우 실망하고 있다. |
| 11 | 어떤 곳에서도 더 이상 즐거움을 느낄 수 없다. |
| 12 | 나는 이제 더 이상 인내할 수 없다. |
| 13 | 나는 새로운 일을 착수할 수 없다. |
| 14 | 도대체 나에게 무엇이 잘못되어 있는가? |
| 15 | 나는 다른 곳에서 살았으면 좋겠다. |
| 16 | 왜 나에겐 모든 것이 뒤죽박죽일까? |
| 17 | 나는 내 자신을 싫어한다. |
| 18 | 나는 가치 없는 인간이다. |
| 19 | 나는 어디론가 사라져 버리고 싶다. |
| 20 | 도대체 나에겐 무엇이 문제란 말인가? |
| 21 | 나는 인생의 패배자다. |
| 22 | 내 인생은 엉망진창이다. |
| 23 | 나는 실패자다. |
| 24 | 나는 결코 성공하지 못할 것이다. |
| 25 | 나는 무기력하다. |
| 26 | 나는 무언가 변화되어야 한다. |
| 27 | 나에겐 틀림없이 무언가 잘못되어 있다. |
| 28 | 나는 미래에 대한 희망이 없다. |
| 29 | 가치 있게 느껴지는 것이 없다. |
| 30 | 나는 어떤 일도 끝까지 해낼 수 없다. |

^4^ Kwon, S, M., & Yun H. (1994) Development and utility of the korean version of the automatic thoughts questionnare. Student Research, 29, 10-25

Korean version of Burnout Assessment (K-BAT)^5^

| 1 | 나는 한 가지 일에 집중하는 것이 힘들다. |
| --- | --- |
| 2 | 나는 명료하게 생각하는 것이 힘들다. |
| 3 | 나는 잘 까먹고 주의가 산만하다. |
| 4 | 나는 집중하기가 힘들다. |
| 5 | 나는 다른 일에 신경 쓰다가 실수를 하곤 한다. |
| 6 | 나는 내 감정을 다스릴 수가 없다. |
| 7 | 내가 감정적으로 어떤 반응을 하는지 인식하지 못한다. |
| 8 | 일이 내가 원하는 대로 흘러가지 않을 경우에 짜증이 난다. |
| 9 | 나는 이유 없이 화가 나거나 슬퍼지곤 한다. |
| 10 | 나는 뜻하지 않게 과하게 반응하곤 한다. |

^5^ Cho, S. (2020). A preliminary validation study for the korean version of the burnout assessment tool (K-BAT). The korean jouranl of industrial and organizational psychology, 33(4), 461-499.
